# Supplementary material for: Fine Particulate Matter and Gaseous Compounds in Kitchens and Outdoor Air of Different Dwellings
Source: Int J Environ Res Public Health. 2020 Jul 21;17(14):5256. doi: 10.3390/ijerph17145256 (PMC7399806; doi:10.3390/ijerph17145256)
Supplement: Supplementary file 1 [file ijerph-17-05256-s001.pdf]

Supplemental Materials for

Fine particulate matter and gaseous compounds in kitchens and outdoor air of different dwellings

Célia Alves[1*], Ana Vicente[1], Ana Rita Oliveira[1], Carla Candeias[2*], Estela Vicente[1], Teresa Nunes[1], Mário Cerqueira[1], Margarita Evtyugina[1], Fernando Rocha[2], Susana Marta Almeida[3]

[1] Centre for Environmental and Marine Studies (CESAM), Department of Environment, University of Aveiro, 3810-193 Aveiro, Portugal

[2] Geobiosciences, Geotechnologies and Geoengineering Research Centre (GeoBioTec), Department of Geosciences, University of Aveiro, 3810-193 Aveiro, Portugal

[3] Centre for Nuclear Sciences and Technologies (C2TN), Instituto Superior Técnico, Estrada Nacional 10, 2695-066 Bobadela, Portugal

*Correspondence to: celia.alves@ua.pt, candeias@ua.pt

Table S1. Statistical comparison between carbonyl concentrations in the kitchens of the four dwellings for a confidence level of 95%. *P*-values of statistically significant differences are in bold.

	House 2	House 3	House 4	Carbonyl
House 1	0.3289	**0.0146**	0.1671	Formaldehyde
	**0.0224**	0.3162	0.0589	Acetaldehyde
House 2		**0.0083**	0.3619	Formaldehyde
		0.0762	0.1479	Acetaldehyde
House 3			0.3619	Formaldehyde
			0.2225	Acetaldehyde

Table S2. Statistical comparison between VOC concentrations in the kitchens of the four dwellings and in the outdoor air for a confidence level of 95%. *P*-values of statistically significant differences are in bold.

Compound	*P*-value
Benzene	0.1183
Toluene	**0.0236**
Tetrachloroethylene	0.6099
Ethylbenzene	**0.0397**
m+p-Xylene	**0.0273**
Styrene	**0.0005**
o-Xylene	**0.0500**
α-Pinene	**0.0001**

Table S3. Statistical comparison of VOC concentrations in the kitchens for a confidence level of 95%. *P*-values of statistically significant differences are in bold.

	House 2	House 3	House 4	Compound
House 1	0.7286	0.1769	**0.0155**	Benzene
	**0.0147**	**0.0449**	0.1512	Toluene
	0.1941	**0.0839**	0.0702	Tetrachloroethylene
	**0.0020**	0.8990	0.1175	Ethylbenzene
	**0.0102**	0.7417	0.1297	m+p-Xylene
	**0.0116**	**0.0384**	0.2776	Styrene
	**0.0112**	0.4352	0.1128	o-Xylene
	0.1890	0.3855	0.6478	α-Pinene
	-	-	-	1,4-Dichlorobenzene
House 2		0.5715	**0.0279**	Benzene
		**0.0330**	0.0684	Toluene
		0.2558	0.3035	Tetrachloroethylene
		**0.0230**	0.0831	Ethylbenzene
		**0.0085**	0.0869	m+p-Xylene
		0.0763	0.1058	Styrene
		**0.0141**	0.0835	o-Xylene
		0.1404	0.2218	α-Pinene
		0.0919	0.0954	1,4-Dichlorobenzene
House 3			**0.0149**	Benzene
			0.0906	Toluene
			0.3727	Tetrachloroethylene
			0.1192	Ethylbenzene
			0.1325	m+p-Xylene
			0.4491	Styrene
			0.1191	o-Xylene
			0.6946	α-Pinene
			0.6358	1,4-Dichlorobenzene

Table S4. Statistical comparison between the $PM_{2.5}$ concentrations in the kitchens of the four dwellings for a confidence level of 95%. *P*-values of statistically significant differences are in bold.

	House 2	House 3	House 4
House 1	**0.0003**	0.6870	0.2086
House 2		**0.0022**	**0.0262**
House 3			0.3344
